# Supplementary material for: The Status of Oxidative Stress in Patients with Alcohol Dependence: A Meta-Analysis
Source: Antioxidants (Basel). 2022 Sep 28;11(10):1919. doi: 10.3390/antiox11101919 (PMC9598131; doi:10.3390/antiox11101919)
Supplement: Supplementary file 1 [file antioxidants-11-01919-s001.zip › antioxidants-1856331-supplementary.pdf]

NEWCASTLE - OTTAWA QUALITY ASSESSMENT SCALE CASE CONTROL STUDIES

Note: A study can be awarded a maximum of one star for each numbered item within the Selection and Exposure categories. A maximum of two stars can be given for Comparability.

Selection

1) Is the case definition adequate?
   a) yes, with independent validation
   b) yes, e.g., record linkage or based on self reports
   c) no description

2) Representativeness of the cases
   a) consecutive or obviously representative series of cases
   b) potential for selection biases or not stated

3) Selection of Controls
   a) community controls
   b) hospital controls
   c) no description

4) Definition of Controls
   a) no history of disease (endpoint)
   b) no description of source

Comparability

1) Comparability of cases and controls on the basis of the design or analysis
   a) study controls for ___Age___ (Select the most important factor.)
   b) study controls for any additional factor ___Gender___ (This criterion could be modified to indicate specific control for a second important factor.)

Exposure

1) Ascertainment of exposure
   a) secure record (e.g., surgical records)
   b) structured interview where blind to case/control status
   c) interview not blinded to case/control status
   d) written self-report or medical record only
   e) no description

2) Same method of ascertainment for cases and controls
   a) yes
   b) no

3) Non-Response rate
   a) same rate for both groups
   b) non respondents described
   c) rate different and no designation

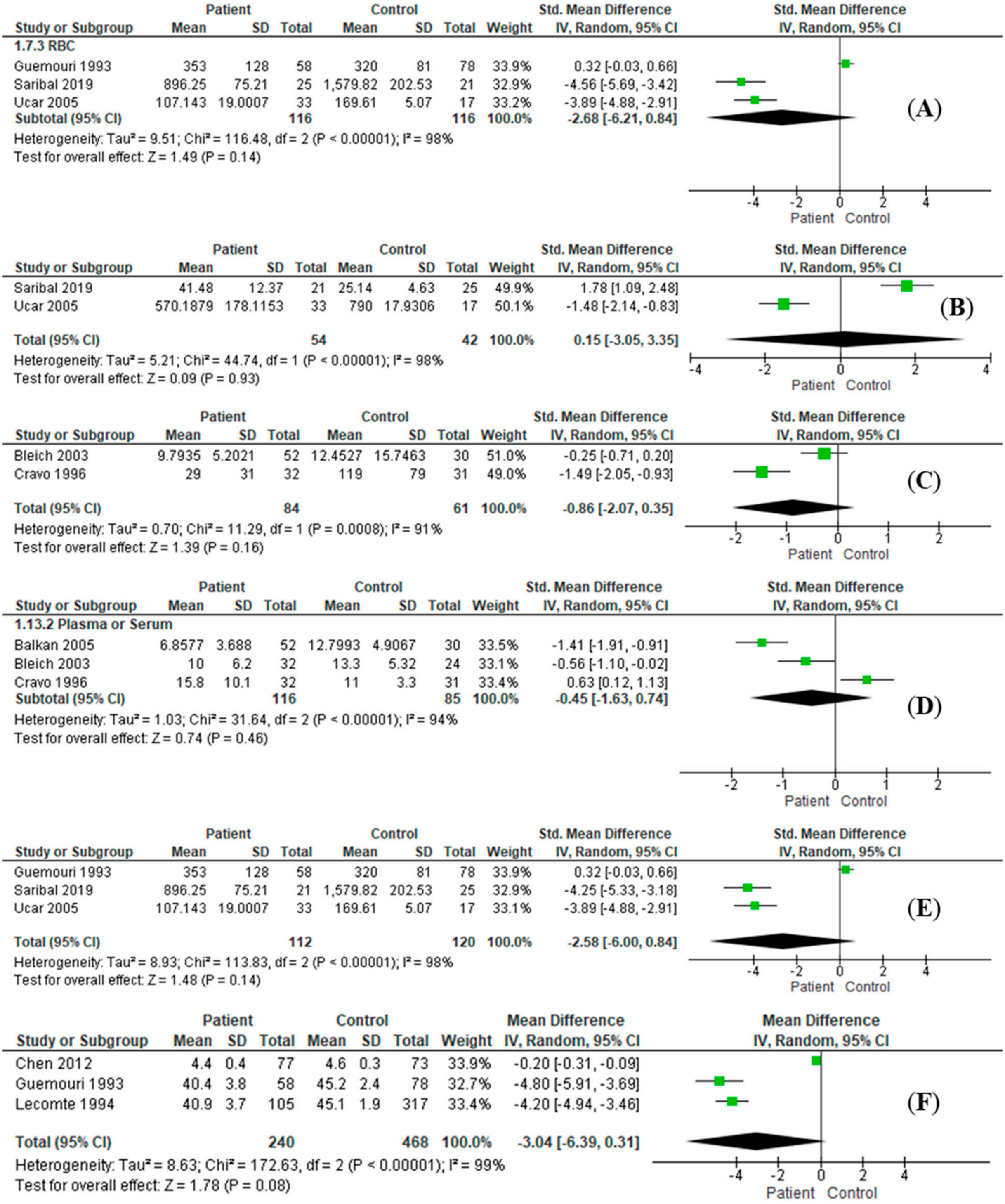

Figure S1. Comparing the levels of antioxidants of alcohol-dependent patients and healthy controls. There were no significant differences found in CAT activity (**A**) and GSH level (**B**) in RBC, or in B6 (**C**) and folic acid (D) in plasma/serum. In male, there were also no significant differences found in CAT activity (**E**) in RBC or albumin (**F**) in plasma/serum between alcohol-dependent patients and healthy

controls. B6, vitamin B6; CAT, catalase; CI, confidential interval; GSH, glutathione; RBC, red blood cells; SD, standard deviation.

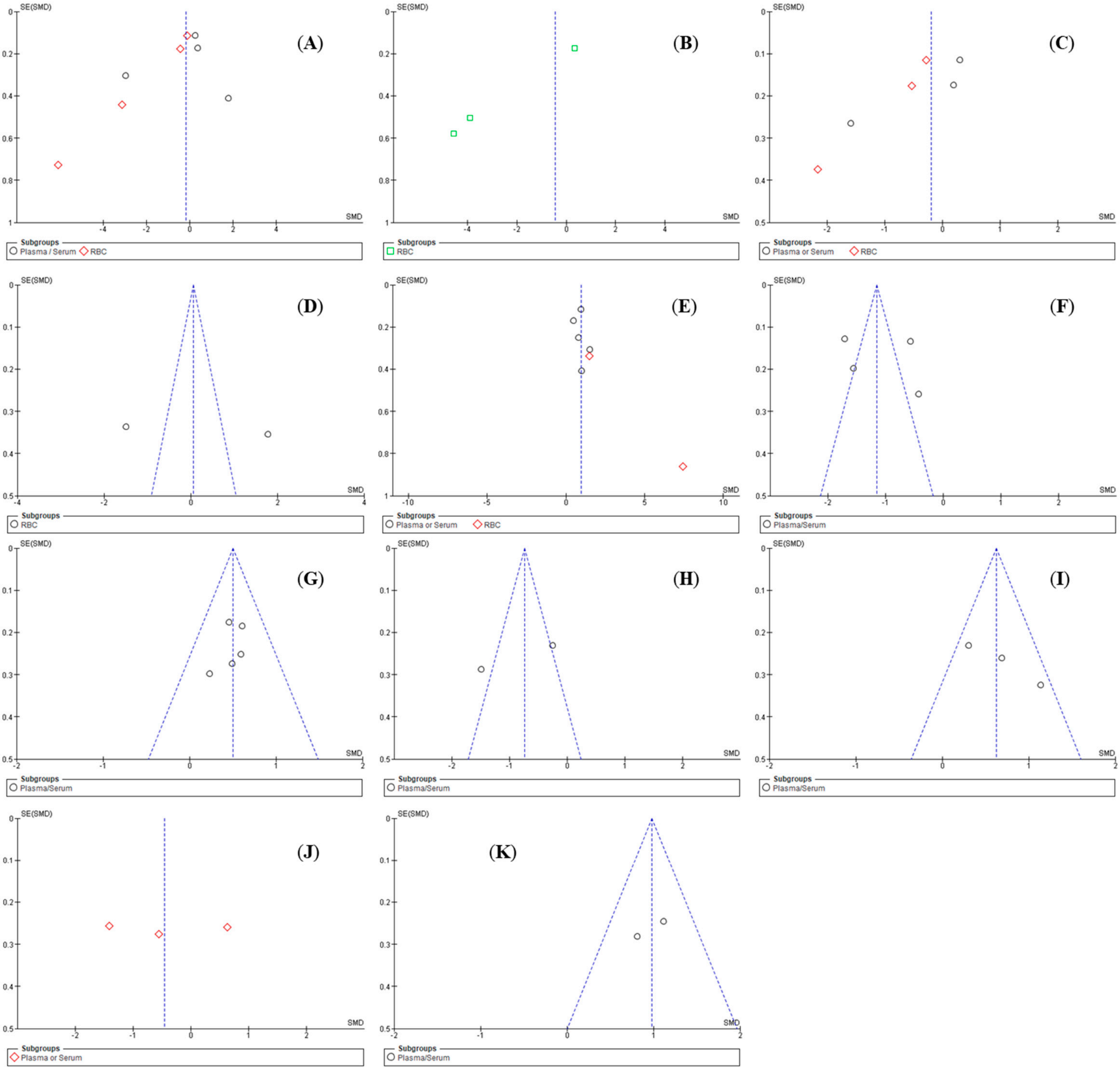

Figure S2. The funnel plot of antioxidants and prooxidants. (**A**) SOD; (**B**) CAT; (**C**) GPx; (**D**) GSH; (**E**) MDA; (**F**) albumin; (**G**) bilirubin; (**H**) B6; (**I**) B12; (**J**) folic acid; (**K**) homocysteine. B6, B12, vitamin B6, B12; CAT, catalase; GPx, glutathione peroxidase; GSH, glutathione; MDA, malondialdehyde; RBC, red blood cells; SE, standard error; SMD, standard mean difference; SOD, superoxide dismutase.

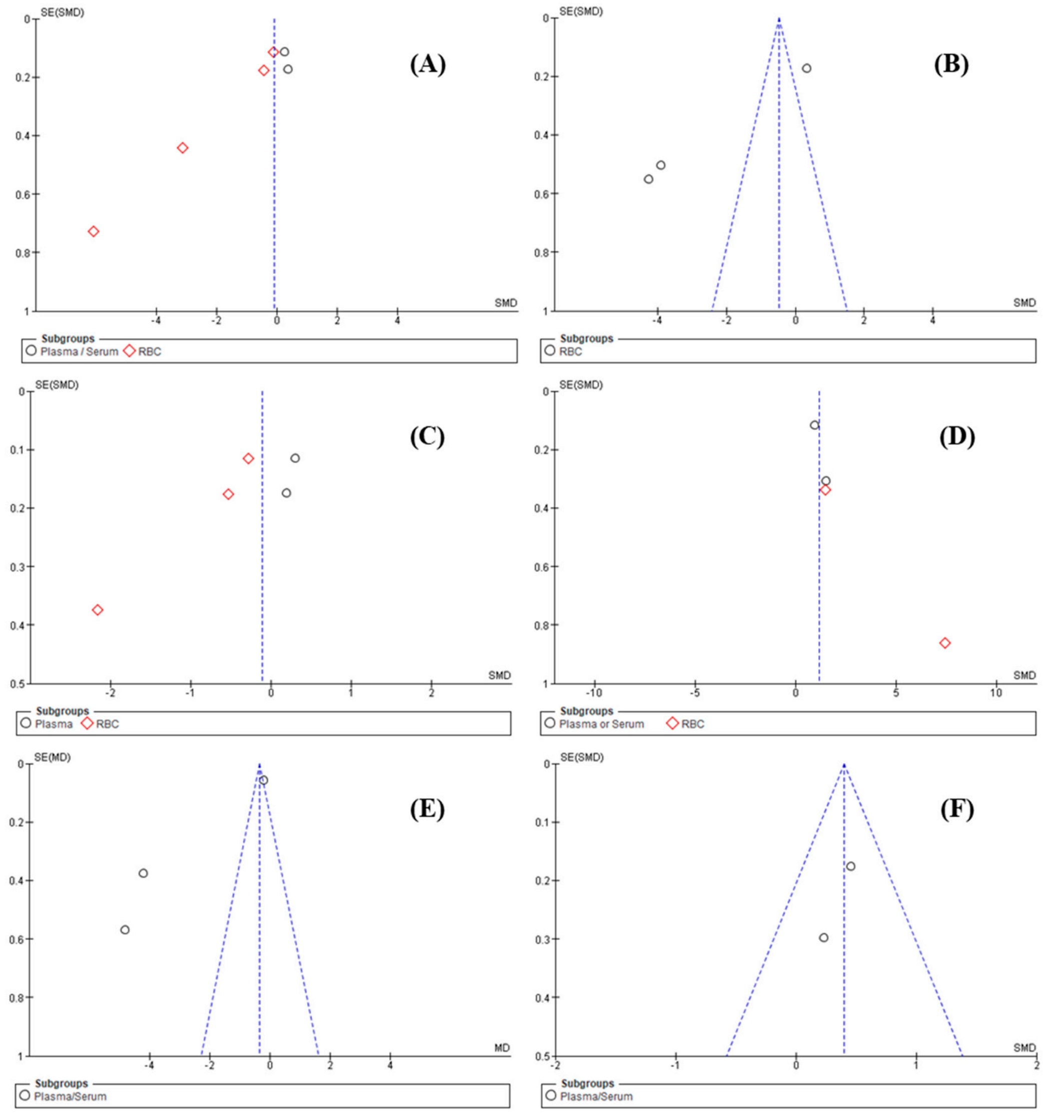

Figure S3. The funnel plot of antioxidants and prooxidants in male. (A) SOD; (B) CAT; (C) GPx; (D) MDA; (E) albumin; (F) bilirubin. CAT, catalase; GPx, glutathione peroxidase; MDA, malondialdehyde; RBC, red blood cells; SE, standard error; SMD, standard mean difference; SOD, superoxide dismutase.

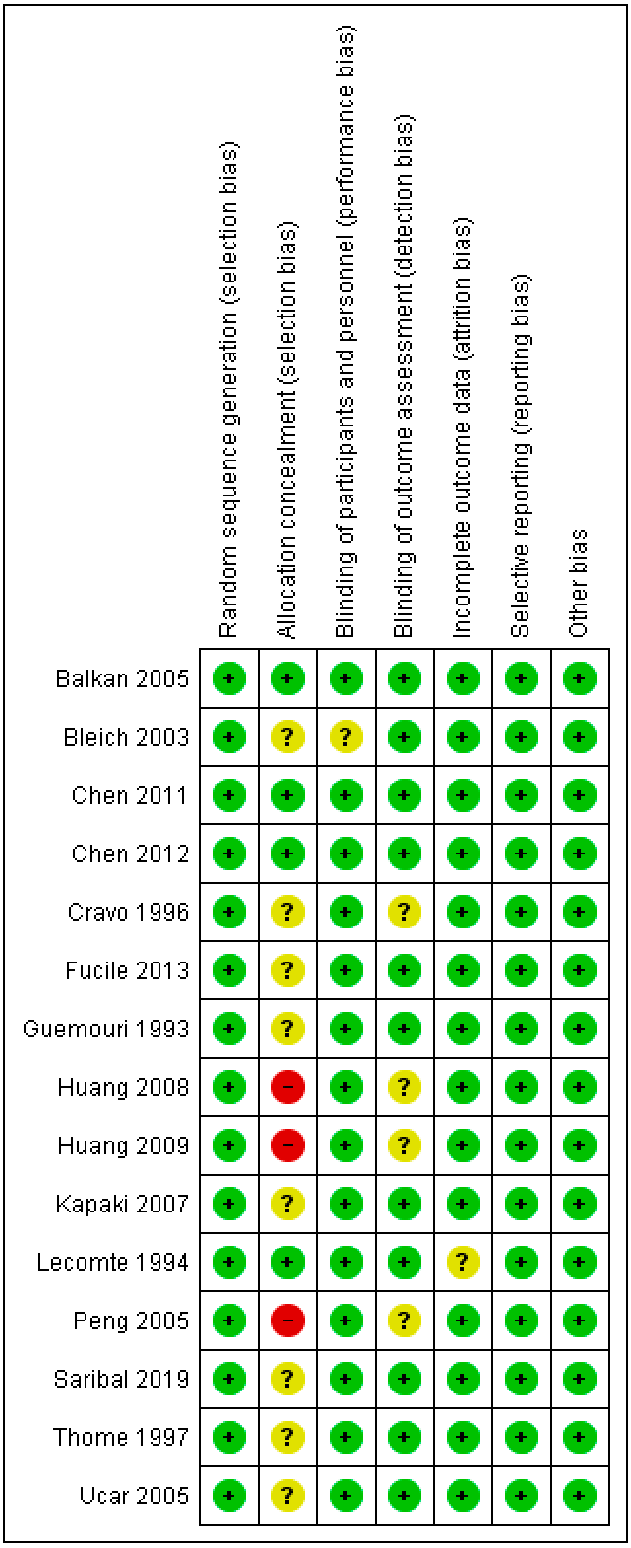

Figure S4. Risk of bias summary: review authors' judgements about each risk of bias item for each included study.

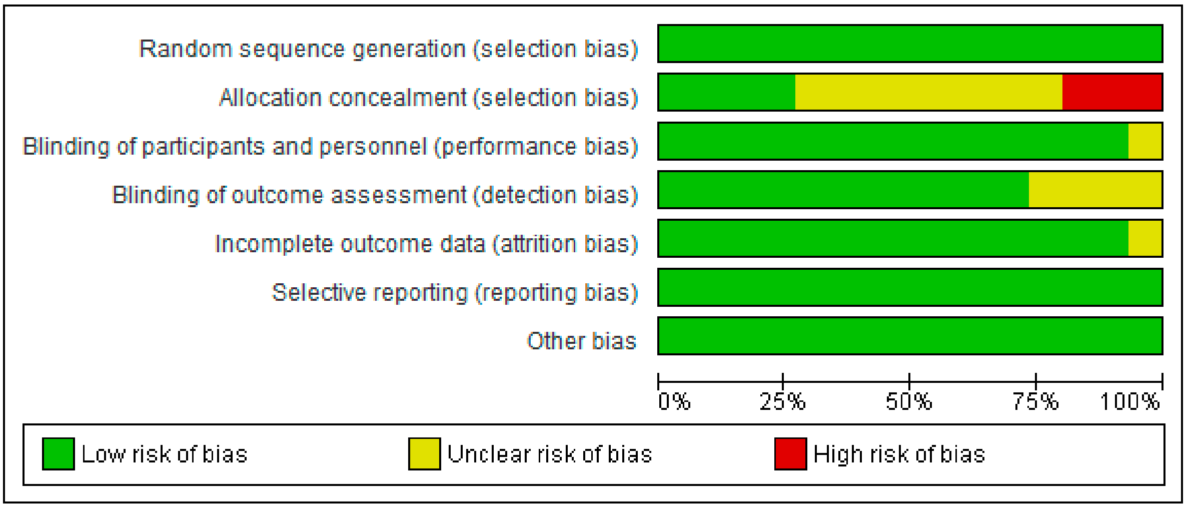

Figure S5. Risk of bias graph: review authors' judgements about each risk of bias item presented as percentages across all included studies.
